# Evolution of miniaturization and the phylogenetic position of *Paedocypris*, comprising the world's smallest vertebrate

**DOI:** 10.1186/1471-2148-7-38

**Published:** 2007-03-13

**Authors:** Lukas Rüber, Maurice Kottelat, Heok Hui Tan, Peter KL Ng, Ralf Britz

**Affiliations:** 1 Department of Zoology, The Natural History Museum, Cromwell Road, London SW7 5BD, UK; 2 Route de la Baroche 12, Case postale 57, CH-2952 Cornol, Switzerland (permanent address) and Raffles Museum of Biodiversity Research, National University of Singapore, Kent Ridge, Singapore 119260; 3 Department of Biological Sciences, National University of Singapore, Kent Ridge, Singapore 119260

## Abstract

**Background:**

*Paedocypris*, a highly developmentally truncated fish from peat swamp forests in Southeast Asia, comprises the world's smallest vertebrate. Although clearly a cyprinid fish, a hypothesis about its phylogenetic position among the subfamilies of this largest teleost family, with over 2400 species, does not exist. Here we present a phylogenetic analyses of 227 cypriniform taxa, including 213 cyprinids, based upon complete mitochondrial DNA cytochrome *b *nucleotide sequences in order to determine the phylogenetic position of *Paedocypris *and to study the evolution of miniaturization among cyprinids.

**Results:**

Our analyses reveal a strongly supported sister group relationship (clade C) between *Paedocypris *and *Sundadanio*, another developmentally truncated miniature cyprinid. Clade C was resolved as sister group of a larger clade characterized by small rasborine taxa (clade D). We found that miniaturised taxa are more numerous in the rasborine clade A, formed by clades C and D, than in any other cyprinid clade. The consensus cyt*b *in cyprinids includes 380 amino acids and an incomplete T–– stop codon. We noted that a few cyprinids mostly rasborine taxa placed within clade A had either a TAA or TAG stop codon, 376, 378, or 381 amino acids, and up to 10 base pairs (bp) of noncoding region before the 5' end of the tRNA-Thr. Our relaxed molecular clock estimates revealed high divergence times for the *Sundadanio *and *Paedocypris *clades and provide a first temporal framework for the evolution of miniaturization among cyprinids.

**Conclusion:**

*Paedocypris *belongs to a clade (Rasborinae clade A) that shows recurrent miniaturization, including both taxa characterized by developmental truncation and by proportioned dwarfism. Its closest relative is another miniaturized taxon, the genus *Sundadanio*. We conclude that the miniaturized cyprinids with remarkable morphological novelties, like *Paedocypris *and *Danionella*, are at the same time the most developmentally truncated taxa. The miniaturized cyprinids with no or few developmental truncations like *Boraras*, *Microrasbora*, and *Horadandia *show no such evolutionary novelties.

## Background

Miniaturisation, an evolutionary process that leads to dwarfed sexually mature organisms, is widespread among vertebrates and best documented in amphibians and fishes [1-4]. Miniaturized taxa are frequently characterized by a trend towards reduction and simplification of various structures and organs. In a number of cases such miniature taxa, in the example of fishes, species maturing at sizes under 20 mm [2], have defied various attempts over a number of decades to determine their phylogenetic position with any confidence. The two most prominent examples among bony fishes in this context are *Schindleria*, which had previously been assigned to various higher level taxa among teleosts and even been put in its own order, until it was shown to be a gobioid [5], and *Sundasalanx*, which was first described as a salmoniform [6], but later demonstrated to be a clupeoid [7].

We recently described a new genus of miniaturized cyprinids, *Paedocypris*, with two new species *P. micromegethes *and *P. progenetica*, both from Southeast Asia [8]. Although clearly a member of the Cyprinidae among the cypriniform Otophysi, the simplified anatomical structure of *Paedocypris*, combined with a number of highly derived autapomorphic characters, have made it difficult to develop a convincing hypothesis about its phylogenetic position among the subfamilies of this largest teleost family [9]. A phylogenetic framework, however, is essential to evaluate the number of evolutionary transitions from non-miniature to miniature among cyprinids, and thus, to determine whether *Paedocypris *is part of a larger group of miniaturized taxa or the consequence of an independent evolutionary event of miniaturization. Among cyprinids, 21 species from nine genera in South and Southeast Asia can be considered miniaturized [8,10-12]. Miniature cyprinids are absent from North America and Eurasia (not including India and southern Asia), however, 12 species in three genera are known from Africa [13]. To determine the phylogenetic position of *Paedocypris *among cyprinids, we performed a phylogenetic analysis based on DNA nucleotide sequence data from a large range of cypriniform representatives including many miniaturized taxa.

## Results

### Cyprinid phylogenetics

The phylogenetic analyses were based on an alignment of 1131 nucleotide sites excluding some positions at the 3' end of the cyt*b *gene. We were unable to amplify the 5' end of the cyt*b *for nine taxa despite designing several new primers located in the tRNA-Glu and internal reverse primers (Additional files 1 and 2). The cytochrome *b *(cyt*b*) in the analysed taxa consists of 376, 378, 380, or 381 amino acids, with 380 amino acid positions being the most common length of the ORF in cyprinids (Additional file 1). Most cyprinids show an incomplete T–– stop codon that is completed to a TAA stop codon posttranscriptionally by polyadenylation of the mRNAs [14]. We noted that a few cyprinids had either a TAA or TAG stop codon and up to 10 base pairs (bp) of noncoding region before the 5' end of the tRNA-Thr. Changes of the 3' end of the cytochrome *b*, the stop codon, noncoding region, and the beginning of the tRNA-Thr of the taxa used in this study are shown in Additional file 1. In this regard it is noteworthy to mention that both *Paedocypris *sp "Pulau Singkep" and "Banka" from Islands near Sumatra show a complete TAA stop codon, whereas *P*. sp "Kalimantan Tengah" and "Pontianak" from Borneo show an incomplete T–– stop codon [see Additional file 1].

**Figure 1:**
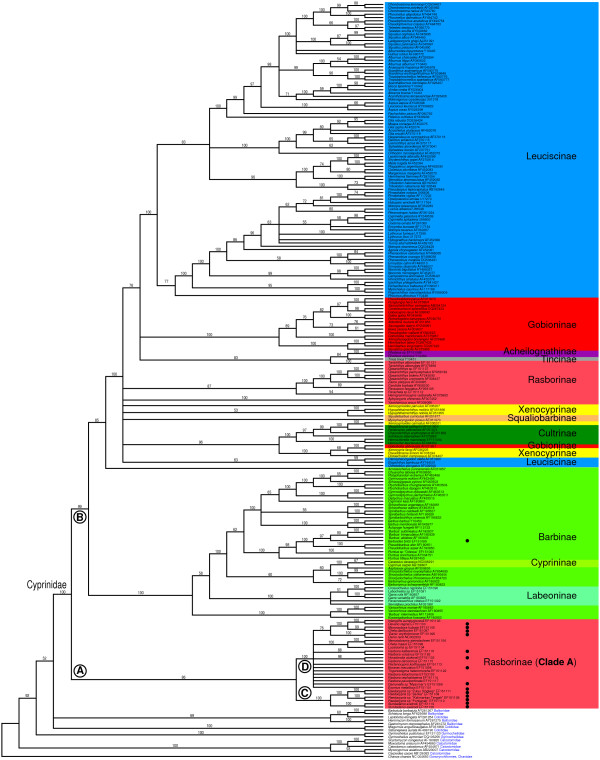
**Phylogeny of the Cyprinidae based upon the complete cyt*b *nucleotide sequence data**. This figure shows the 50% majority rule consensus tree of 5000 MC3 sampled trees. Major clades discussed in the text are labelled A to D. Bayesian posterior probabilities (in percentage) are given above branches. Assignment of taxa to the eleven cyprinid subfamilies follows [24] with modifications by [9, 47] (subfamilies are illustrated with colour boxes, see Additional file 1). A black filled circle next to the species name indicates miniature taxa.

The 50% majority-rule consensus tree recovered from the partitioned Bayesian analysis of the complete cytochrome *b *is depicted in Figure 1. As the focus of our study was to place *Paedocypris *within a larger phylogenetic framework, a detailed treatment of cyprinid intrarelationships based upon complete cyt*b *is beyond our scope. We are aware that some nodes in Figure 1 are poorly supported or unresolved but this does not affect our conclusions. Clearly, more nucleotide sampling both from mitochondrial and nuclear DNA is needed to fully resolve the complex phylogenetic history of cyprinids.

**Figure 2:**
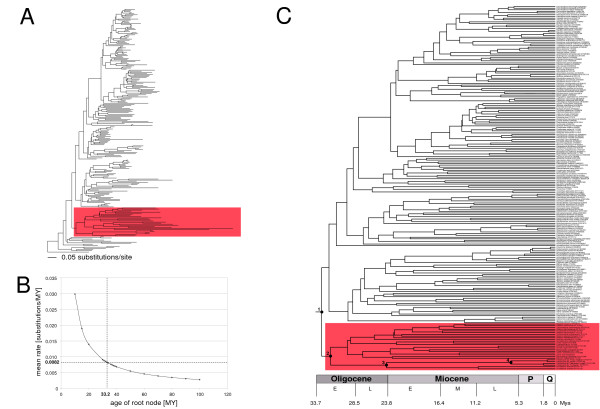
**Relaxed molecular clock analysis of cyprinids based on complete cyt*b *nucleotide sequence data**. Relaxed molecular clock for cyprinids based on penalized likelihood (PL) and a calibration using a mean substitution rate of 0.0082 substitutions per site per million year (see text for details). A) ML phylogram based upon the complete cyt*b *nucleotide sequence data. B) Relationship between root age and inferred mean substitution rate for the cyprinid tree. The tree was calibrated by iteration of the age of the root [44] until the mean rate equaled the cyprinid cyt*b* clock [16] For each root age the optimal smoothing value for PL was assessed independently using the cross validation procedure implemented in r8s. A root age of 33.2 MY resulted in a mean substitution rate of 0.0082 substitutions per site per million year [16]. C) Resulting chronogram using a fixed root age of 33.2 MY. The nodes labelled 1–4 are mentioned in the text and in Table 1. The Rasborinae clade A (see Figure 1) is highlighted in both A and C.

*Paedocypris *was placed as sistergroup of *Sundadanio *with high support (1.0 posterior probability, clade C in Figure 1). The *Paedocypris-Sundadanio *clade C was resolved with moderate support as sistergroup to clade D (Figure 1; 1.0 posterior probability), which consists of an additional 21 rasborine taxa forming the Rasborinae clade A (0.86 posterior probability, clade A in Figure 1). Other taxa commonly classified as Rasborinae were not resolved in clade A, rendering the Rasborinae non-monophyletic (Figure 1). The Rasborinae clade A was resolved as sister group of clade B (Figure 1; 0.99 posterior probability) consisting of the remaining cyprinid taxa with high support. The monophyly of the Cyprinidae received a posterior probability of 1.00 (Figure 1). ML gave identical results regarding the main cyprinid intrarelationships as BI (see Figure 2). The maximum observed pairwise genetic distance (p-distances) between *Paedocypris *individuals sampled from Sumatra and Borneo were 12.6%, whereas the genetic distance between the two *Sundadanio *individuals (one from Borneo and the other with uncertain origin obtained through the aquarium trade, but presumably from Sumatra) was 13.5%.

### Cyprinidae divergence time estimates

The ML phylogram used for the PL analyses is depicted in Figure 2A. In both approaches, calibration based on substitution rate or fossil record, stem and crown group calibrations gave nearly identical results regarding cyprinid divergence time estimates (Table 1). We therefore focus on the results obtained using the crown group calibration. Applying the cyprinid cyt*b *molecular clock of 0.0082 substitutions per site per million year resulted in a cyprinid crown group age of 33.2 MY (node 1 in Figure 2C), while the age of the MRCA of rasborins (Clade A) was 31.89 MY (node 2 in Figure 2C), that of *Paedocypris *and *Sundadanio *was 23.97 MY (node 3 in Figure 2C), and that of *Paedocypris *was 6.34 MY (node 4 in Figure 2C) (Table 1). The inferred standard deviation (sd) of the substitution rate was 0.003. In contrast, when the cyprinid tree was calibrated with fossil data constraining the crown group age to 51.9 MY (node 1 in Figure 2C), the MRCA of rasborines (Clade A) was found to be 49.86 MY old (node 2 in Figure 2C), that of *Paedocypris *and *Sundadanio *37.47 MY (node 3 in Figure 2C), and that of *Paedocypris *9.91 MY (node 4 in Figure 2C), respectively (Table 1). The inferred mean substitution rate with this calibration was 0.0052 (sd 0.002) substitutions per site per million years.

**Table 1:** Molecular divergence time estimates of selected cyprinid nodes.

Taxa	Substitution rate calibration		Fossil calibration	
		
	Crown group	Stem group	Crown group	Stem group
MRCA Cyprinidae and its sister group	n.a	34.20	n.a	51.90 ^a^
MRCA Cyprinidae	33.20	33.19	51.9 ^a^	50.38
MRCA Rasborinae (clade A)^b^	31.89	31.89	49.86	48.42
MRCA *Paedocypris *and *Sundadanio *(clade C)^b^	23.97	23.99	37.47	36.41
MRCA *Paedocypris*	6.34	6.36	9.91	9.67
				
Substitution rate	0.0082^c^	0.0082^c^	0.0053^d^	0.0054^d^
Standard deviation (sd)	0.0032	0.0032	0.0021	0.0021
Smoothing parameter for PL	1.0	1.0	2.5	2.5

This table lists inferred ages in million years for selected cyprinid nodes (nodes MRCA Cyprinidae, MRCA Rasborinae (clade A), MRCA *Paedocypris *and *Sundadanio*, and MRCA *Paedocypris *are labelled 1–4 in Figure 2C), substitution rates, standard deviation and smoothing parameters used for the alternative calibration of a relaxed cyprinid molecular clock (substitution rate calibration and fossil calibration) with penalized likelihood (PL). The chronogram resulting from the substitution rate calibration (crown group) is shown in Figure 2C.

^a ^= fixed root age; ^b ^= Clades A and C refer to clade designations given in Figure 1; ^c ^= fixed substitution rate (see text for details); ^d ^= inferred substitution rate.

### Evolution of miniaturization in rasborines

**Figure 3:**
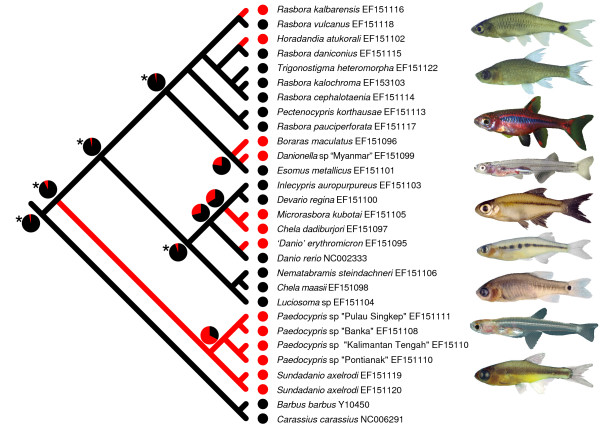
**Evolution of miniaturization in the rasborine clade A**. Tracing the evolution of miniaturization on the ML topology (one of three ML topologies; choice of topology does not alter conclusions) of the rasborine data set (clade A). The state 'miniature' is given in red colour, whereas the state 'non-miniature' is given in black. Results from both, unweighted parsimony and ML (selected nodes only), are shown. Significant ML reconstructions are indicated with an asterisk. Photographs of selected miniature rasborines mentioned in the text are given behind taxon names: *Rasbora kalbarensis*, *Horadandia atukorali*, *Boraras brigittae*, *Danionella *sp 'Myanmar', *Microrasbora kubotai*, *Chela dadiburjori*, *'Danio' erythromicron*, *Paedocypris progenetica*, *Sundadanio axelrodi*.

The ML topology from the rasborine data set was used to perform character-state reconstruction for the evolution of miniaturization among Rasborinae clade A (Figure 1). Using both, unweighted parsimony and ML ancestral character state reconstruction we found that a miniature body size has evolved recurrently among rasborines from clade A (Figure 3). We note, however, that the topology of clade D is not well resolved (Figure 1) and that additional mitochondrial and nuclear DNA sequence data as well as an increased taxon sampling is needed for a better understanding of the evolution of miniaturization in rasborine clade A.

## Discussion

### Molecular phylogenetics

To place *Paedocypris *within a larger phylogenetic framework we had to depend to a large degree on published cyt*b *sequences, by far the most commonly used molecular phylogenetic marker for cyprinids thus far (e.g. [15-18]). While most of the cyprinid subfamilies are rather well represented in the GenBank/EMBL/DDBJ database, complete rasborine cyt*b *sequences are scarce. This is surprising, since the Rasborinae is a particularly speciose and widespread subfamily. Only one representative of the rasborine clade A (Figure 1) could be obtained from GenBank (*Danio rerio*, NC 002333) whereas the other 26 species (clade A) were sequenced specifically for this study. We were unable to amplify the 5' end of the cyt*b *for nine of these taxa [see Additional file 1]. It is therefore possible that in some rasborines the tRNA-Glu is not located directly 5' of the cyt*b *or that it shows an unusual structure compared to the consensus teleost tRNA-Glu. This might partially explain the absence of published complete rasborine cyt*b *nucleotide sequences.

### Divergence time estimates

The results derived from the substitution rate calibration and those obtained under the fossil calibrations vary widely in the inferred cyprinid root ages (Table 1). While the root was fixed at an age of 51.9 My based on fossil evidence, using the substitution rate calibration we obtained a root age of 33.2 My (Table 1). A possible source of error in this calibration is the cyprinid cyt*b *substitution rate used. Recently, it was shown that there is a problem of extrapolating molecular rates across different evolutionary timescales caused by marked differences between short-term and long-term substitution rates [19]. It is therefore likely that the cyprinid substitution rate we employed is inflated (faster short term substitution rates for the taxa used for the calculation [16] than the "real" long term cyprinid substitution rate) and hence, the divergence time estimates have been underestimated.

### Miniaturization

The evolution of small size is a recurrent theme among teleosts and especially striking in cyprinid and gobioid fishes, which include the record holders for smallest vertebrates [8,20,21]. The recently described cyprinid genus *Paedocypris *contains the smallest fish and vertebrate species, *P. progenetica*, with females maturing at 7.9 mm standard length [8]. So far, the phylogenetic relationships of *Paedocypris *among the Cyprinidae are unclear due to its highly developmentally truncated anatomy. The mtDNA based phylogenetic analyses, reported herein are part of a dual approach to resolve this issue. An ongoing morphological study looks at non-truncated characters of *Paedocypris *and compares them with other cyprinid representatives (Britz and Conway in prep.). Our phylogenetic analyses of a large number of cyprinids consistently recovered *Sundadanio *as the sister group of *Paedocypris*. *Sundadanio *is a genus established for a single miniature species, *S. axelrodi*, originally described as a member of the genus *Rasbora *[10,22]. *Sundadanio *(which includes at least two or three undescribed species) has a maximum size of 22.5 mm SL [23] and occurs on Sumatra, Banka, Riau Archipelago, and different parts of Borneo.

Both *Sundadanio *and *Paedocypris *are part of a larger clade (Rasborinae clade A in Figure 1) comprising taxa that have been referred to in the systematic literature as Rasborinae [24] or Danioninae [25]. Among those, the genera *Esomus*, *Luciosoma*, *Rasbora*, *Nematabramis*, *Chela*, *Inlecypris*, *Danio *(= *Devario*), *Brachydanio *(= *Danio*), *Bengala*, *Pseudorasbora*, and possibly *Thryssocypris*, were considered by Howes [24] to form a monophyletic group, the Rasborini. The only more recent comprehensive phylogenetic study looking at relationships among some rasborine taxa is Fang [26], who focused on the so-called danionins. Regarding the position of *Sundadanio*, Fang ([26], p. 719) concluded: "The genus is obviously well characterized, and it is apparently a danionin taxon, but a precise phylogenetic placement is presently elusive." Based on our molecular results *Sundadanio *is the closest relative of *Paedocypris*.

Judging from the number of very small species, miniaturization seems to be much more frequent in the rasborine clade A cyprinids than in any other subfamily (Figure 1). In the taxa we have studied, miniaturization occurs in the *Sundadanio*/*Paedocypris *clade (clade C: Figure 1), in *Danionella*, '*Danio' erythromicron*, *Microrasbora*, *Horadandia*, *Boraras*, *Chela dadiburjori *and in *Rasbora kalbarensis*. Based on our tree, we hypothesize that miniature taxa evolved at least seven times independently in rasborine clade A (Figure 3). The only other miniature cyprinid outside Rasborinae in our analysis is the African *Barboides britzi*, which groups with the other African *Barbus*-like cyprinids (Figure 1). Other miniature cyprinid taxa, which we were unable to include in the study, are *Sawbwa resplendens*, an Asian member of the Cyprininae, *Tanichthys micagemmae*, sometimes considered a rasborine, although its sister taxon *T. albonubes *was not resolved in the core rasborine clade A in our analyses (Figure 1), and nine African species of the genus *'Barbus' *(Barbinae).

Our results indicate that *Paedocypris *is the sister genus of the miniaturized *Sundadanio *and part of a larger clade, the Rasborinae clade A (Figure 1). *Paedocypris *is thus not the result of an independent miniaturization event, but an extreme of the trend towards miniaturization in the *Sundadanio *– *Paedocypris *clade (Figure 3). Although miniature fishes, by definition, share the character 'maturing at sizes under 20 mm' [2], the anatomical outcome of the process of miniaturization can be very different. Two extreme results are possible with various intermediate stages in between [27]: the miniaturized species may just be a dwarfed but otherwise identical image of its larger ancestor (Gould's proportioned dwarfism [28]), or it closely resembles an early developmental stage of the larger ancestor (commonly referred to as developmentally truncated species). Among the miniature cyprinids, an example for the first case is *Boraras*, which, except for a few reductions, closely resembles its larger relatives of the genus *Rasbora *[29]. Two clearly developmentally truncated miniature cyprinids are *Danionella *and *Paedocypris *that have the appearance and anatomical structure of larval cyprinids [8,11].

Hanken [1] noted that miniaturization is often not only associated with the reduction of characters, but also with the evolution of morphological novelties and some of the developmentally truncated miniature cyprinids offer fascinating examples for this claim. *Danionella *is characterized by an anterior shift of the genital pore and anus in males so that both open between the enlarged pelvic fins [11], and by novel flanges, cartilages, and processes on the Weberian apparatus with unknown function. Males of the genus *Paedocypris *have a highly modified pelvic girdle, and pelvic fin rays and associated muscles that along with a conspicuous, keratinized knob of skin in front of the fins possibly function as a clasping organ, although its precise biological role is still unclear [8]. In contrast, species of the genus *Boraras*, the proportioned dwarfs, which are in roughly the same size class as *Danionella *and *Paedocypris*, reveal no such evolutionary novelties. Morphological novelties also seem to be lacking in some of the other miniaturized, but hardly developmentally truncated, cyprinids, like *Horadandia*, *Sawbwa*, *Microrasbora*, and '*Danio' erythromicron*. With the establishment of *Danio rerio*, a member of rasborine clade A, as a model organism for vertebrate developmental genetics [30] the proportioned dwarfs and the developmentally truncated miniatures offer a challenging system of "natural mutants" to study the loss of characters and the evolution of morphological novelties comparatively at a genetic level.

Kottelat et al. [8] pointed out that peat swamp forests in Southeast Asia house an unusually high number of miniature fishes. Regarding cyprinids only, this still holds true, as six out of the 12 miniature cyprinids occurring in Southeast Asia live in peat swamp forests and of these five exclusively so (i.e. are stenotopic). The continued study of these and other miniaturized fish is in jeopardy as their preferred habitats in Southeast Asia are being lost at an alarming rate [8].

## Conclusion

Our phylogenetic analyses that include representatives of all major cyprinid lineages show a strongly supported sister group relationship between *Sundadanio *and *Paedocypris*, two developmentally truncated taxa. They were resolved as part of a larger clade containing small rasborines (rasborine clade A). Relaxed molecular clock analyses revealed unexpectedly old ages for the MRCAs of the *Sundadanio *– *Paedocypris *clade (clade C; Figure 1) and the *Paedocypris *and *Sundadanio *clades, respectively (Figure 1 and 3). Miniaturization seems to be a much more frequent event in the rasborine clade A than in any other cyprinid subfamily. Based on our phylogenetic hypothesis, miniature taxa evolved at least seven times independently in rasborine clade A including developmentally truncated taxa and taxa characterized by proportioned dwarfism. The rasborine clade A is not only an ideal group to study the evolution of miniaturization among vertebrates, but also to investigate the evolution of morphological novelties. While those miniature cyprinids that hardly show any developmental truncation generally lack morphological novelties, they are common in miniature, developmentally truncated cyprinids.

## Methods

### Biological material, DNA isolation, and DNA sequencing

To assess the molecular phylogenetic position of *Paedocypris*, DNA samples of 36 Cyprinidae, mostly Rasborinae, and one species of Gyrinocheilidae were specifically obtained for this study (appendix 1; see below). In addition, a total of 191 complete or nearly complete cytochrome *b *(cyt*b*) sequences were obtained from GenBank (177 Cyprinidae, five Catostomidae, three Cobitidae, four Balitoridae, one species of Gyrinocheilidae, and one species of Gonorynchiformes as outgroup; see Additional file 1). Cytochrome *b *has been the most important and most frequently used molecular marker in cyprinid phylogenetics, but we are aware that its phylogenetic performance might not be suitable to address cyprinid intrarelationships at all taxonomic levels [31]. However, we still chose to use cyt*b *over alternative markers due to its huge taxonomic coverage that allowed wide ranging comparisons regarding the phylogenetic position of *Paedocypris*.

Whole fish or fin clips were preserved in 70–100% ethanol, and total genomic DNA was isolated from white muscle tissue or fin clips using the QIAGENE DNeasy Tissue kit. The complete cytochrome *b *gene was amplified with two versatile primers DonGlu F and DonThr R [32]. For some taxa additional primers were used [see Additional file 2]. All PCR amplifications were conducted in 25 μl reactions containing 75 mM Tris-HCl (pH 9.0), 2 mM MgCl2, 0.4 mM of each dNTP, 0.4 μM of each primer, template DNA (10–100 ng), and Taq DNA polymerase (1 unit, Promega), using the following program on a MJ PTC-2000 thermal cycler: 1 cycle of 2 min at 94°C, 35 cyles of 60s at 94°C, 60s at 48–54°C, and 90s at 72°C, and finally, 1 cycle of 5 min at 72°C. PCR products were sequenced directly after PCR purification using the Millipore PCR cleanup kit.

Sequencing reactions were performed with the BigDye Terminator v1.1 Cycle Sequencing Kits (Applied Biosystems, Foster City, CA) following manufacturer's instructions in a 10 μl volume with 1 pMol of primer, 1 μl of BigDye Terminator Mix, and 2–3 ng of DNA per 100 bps of PCR product. The cycling profile for the sequencing reaction consisted of 25 cycles of 10 s at 96°C, 5 s at 50°C, and 4 min at 60°C. Cycle sequencing products were purified using standard ethanol/sodium acetate precipitation and run on an Applied Biosystems 3730 × l DNA Analyzer. Sequences specifically obtained for this study have been deposited in GenBank [GenBank: EF151088–EF151123 and EF153103 ].

### Sequence alignment and phylogenetic analyses

The cytochrome *b *nucleotide data set was aligned by eye. The alignment is available from TreeBASE. The phylogenetic analyses comprised the complete cyt*b *of 228 taxa (including other families and outgroup; Appendix 1). The Akaike Information Criterion (AIC; [33]) implemented in MODELTEST v3.06 [34] was used to determine the evolutionary model that best fits the data set. The model selected was subsequently used for Bayesian inference (BI) and maximum likelihood (ML) analyses.

A Bayesian inference (BI) of cyprinid phylogeny was performed with MrBayes v3.1.2 [35] by Metropolis Coupled Markov Chain Monte Carlo (MC3) sampling for 2,000,000 generations (two independent runs each with four simultaneous MC chains; chain temperature 0.2; sample frequency 200; burnin 1,500,000 generations (see Results) under the GTR + I + Γ model as selected by MODELTEST v3.06. The cyt*b *data set was run with three data partitions (1st, 2nd, and 3rd codon positions) and model parameters were estimated independently for each of the respective data partitions using the unlink command in MrBayes v3.1.2. Tracer v1.3 [36] was used to plot the – log likelihood scores against generation time to evaluate mixing, run convergence, and the burn-in needed before reaching stationarity. We then used PAUP* v4.0b10 [37] to reconstruct the 50% majority-rule consensus tree of the post burn-in trees. ML analyses were conducted with Garli v0.94 [38] under the GTR + I + Γ model and using the default settings.

### Evolution of miniaturization

Ancestral character state reconstructions for the evolution of miniaturization in the rasborine clade A were performed based upon ML topology obtained with PAUP* from a restricted 29-taxon data set (henceforth referred to as the rasborine data set). Miniaturized taxa are defined as maturing at sizes under 20 mm [2]. Ancestral character state reconstruction was performed under unweighted parsimony and ML as implemented in Mesquite v1.06 [39,40].

### Divergence times estimates

Chronograms were constructed using penalized likelihood (PL, [41]), as implemented in r8s v1.70 [42] based on the ML phylogram to date major cladogenetic events. The TN algorithm and the additive penalty function was used for the PL analyses. In order to find the optimal smoothing parameter (λ) for PL, cross-validation was performed over a range of values of λ ranging from 10^0^ to 10^2.8 ^in 15 steps.

To roughly estimate divergence times between clades of interest we used two approaches. The first approach does not rely on the fossil record, but instead assumes an average cyprinid cyt*b *substitution rate of 0.0082 substitutions per site per million years. This substitution rate was derived for the same gene for European cyprinids based upon two independent, and well-dated geological events (formation of the strait of Korinthos and the opening of the Gibraltar strait after the Messinian salinity crisis) [16] and was recalculated by Rüber et al. [43]. An iterative approach was used to estimate divergence times for the cyprinid data set by adjusting the age of the cyprinid root (see below) until it fitted the average cyprinid substitution rate (see also [44]). The second approach makes use of the fossil record of cyprinids. The oldest known fossil of the Cyprinidae is *Parabarbus *sp. from the Early Eocene (Ypresian, 49.0 – 54.8 MYA; Obailinskaya formation in Kazakhstan; [45,46]). We used the median age of the Ypresian (51.9 MYA) to calibrate the cyprinid tree. Given the uncertainty of assigning *Parabarbus *sp. to either the stem or crown group Cyprinidae, we conducted both analyses using either the most recent common ancestor (MRCA) of cyprinids and its sister group or the MRCA of cyprinids as the fixed "cyprinid root", respectively.

## Abbreviations

BI, Bayesian inference; bp, base pairs; cyt*b*, cytochrome *b*; MC3, Metropolis Coupled Monte-Carlo-Markov-Chains; ML, maximum likelihood; MRCA, most recent common ancestor; MY, million years; MYA, million years ago; PL, penalized likelihood; sd, standard deviation.

## Authors' contributions

All authors designed the study. MK and HHT were involved in sampling. LR carried out the molecular work and the analyses. All authors contributed to the preparation of the manuscript, RB is responsible for the parts dealing with miniaturization. They read and approved the final version.

## Supplementary Material

Additional File 1 **Table 1 – Specimen information, GenBank accession numbers, and alignment of the 3' end of the cyt*b*, stop codon, non coding region, and 5' end of tRNA-Thr of the taxa included in this study**. This table lists the families, subfamilies, species, and GenBank accession numbers (including sequences directly obtained from GenBank; GenBank entries from this study are underlined) for the cyt*b *nucleotide sequence data. Subfamily assignments of the cyprinid taxa follow [24] with modifications from [9,47]. The alignment of the 3' end of the cyt*b*, stop codon, non coding region, and 5' end of tRNA-Thr is given to illustrate changes occurring in this nucleotide region (non coding region, and 5' end of tRNA-Thr are not shown for taxa with a complete mitochondrial genome entry in GenBank). Click here for file

Additional File 2 **Table 2 – Additional primers used to amplify the complete cyt*b *of the taxa sequenced specifically for this study**. This table lists the additional primers used for this study. For previously published primers the references are given, primers designed for this study are given 5' to 3'. Click here for file
