# Supplementary material for: Evolution of miniaturization and the phylogenetic position of Paedocypris, comprising the world's smallest vertebrate
Source: BMC Evol Biol. 2007 Mar 13;7:38. doi: 10.1186/1471-2148-7-38 (PMC1838906; doi:10.1186/1471-2148-7-38)
Supplement: Additional File 1 — Table 1 – Specimen information, GenBank accession numbers, and alignment of the 3' end of the cytb, stop codon, non coding region, and 5' end of tRNA-Thr of the taxa included in this study. This table lists the families, subfamilies, species, and GenBank accession numbers (including sequences directly obtained from GenBank; GenBank entries from this study are underlined) for the cytb nucleotide sequence data. Subfamily assignments of the cyprinid taxa follow [24] with modifications from [9,47]. The alignment of the 3' end of the cytb, stop codon, non coding region, and 5' end of tRNA-Thr is given to illustrate changes occurring in this nucleotide region (non coding region, and 5' end of tRNA-Thr are not shown for taxa with a complete mitochondrial genome entry in GenBank). [file 1471-2148-7-38-S1.PDF]

Additional file 1

Family	Subfamily	Genus	Species	Accession	3' end of cyt*b* and adjacent region[1]
Cyprinidae	Acheilognathinae	*Rhodeus*	*sericeus*	Y10454	GCATTAAAATGAGCT---N------------NNNNNNNNNN
Cyprinidae	Acheilognathinae	*Rhodeus*	sp	EF151088	GCGCTAAAATGAGCT---T------------GCACTAGTAG
Cyprinidae	Barbinae	*Aaptosyax*	*grypus*	AF309636	ACACTGAAATGAGCT---N------------NNNNNNNNNN
Cyprinidae	Barbinae	*Aulopyge*	*huegelii*	AF112133	GCACTCGAATGAGCT---N------------NNNNNNNNNN
Cyprinidae	Barbinae	*Barboides*	*britzi*	EF151089	GCACTTAAA---------TAATTT-------GCACTAGTAG
Cyprinidae	Barbinae	*Barbonymus*	*gonionotus*	AF180822	GCACTAGAATGAGCT---N------------NNNNNNNNNN
Cyprinidae	Barbinae	*Barbonymus*	*schwanenfeldii*	AF180823	GCACTAGAATGAGCT---N------------NNNNNNNNNN
Cyprinidae	Barbinae	*'Barbus'*	*ablabes*	AF180835	ACACTTAAATTAACC---N------------NNNNNNNNNN
Cyprinidae	Barbinae	*Barbus*	*barbus*	Y10450	GCACTAGAATGAGCT---N------------NNNNNNNNNN
Cyprinidae	Barbinae	*'Barbus'*	*intermedius*	AF112406	GCACTAGAATTAACC---N------------NNNNNNNNNN
Cyprinidae	Barbinae	*Barbus*	*meridionalis*	AF045977	GCACTAGAATGAGCT---T------------NNNNNNNNNN
Cyprinidae	Barbinae	*'Barbus'*	*sublineatus*	AF180837	ACACTCAAATTAAAC---N------------NNNNNNNNNN
Cyprinidae	Barbinae	*'Barbus'*	*trimaculatus*	AF180839	ATACTAAAC---------TAATTT-------NNNNNNNNNN
Cyprinidae	Barbinae	*Chuanchia*	*labiosa*	AY608650	GCACTGGAATTAGCC---N------------NNNNNNNNNN
Cyprinidae	Barbinae	*Crossocheilus*	*nigriloba*	EF151090	GCACTAAAATGAGCT---T------------GCCCTAGTAG
Cyprinidae	Barbinae	*Cyprinion*	*kais*	AF180860	GCACTAGAATGAGCT---N------------NNNNNNNNNN
Cyprinidae	Barbinae	*Diptychus*	*maculatus*	AY463515	GCACTGGAATTAGCC---N------------NNNNNNNNNN
Cyprinidae	Barbinae	*Garra*	*rufa*	AF180857	GCTCTACAA---------TAA----------GCTNNNNNNN
Cyprinidae	Barbinae	*Garra*	*variabilis*	AF180825	GCACTAGAATGAGCT---N------------NNNNNNNNNN
Cyprinidae	Barbinae	*Gymnocypris*	*eckloni*	AY463494	GCATTAGAATTAGCC---N------------NNNNNNNNNN
Cyprinidae	Barbinae	*Gymnodiptychus*	*dybowskii*	AY463513	GCACTGGAATTAACC---N------------NNNNNNNNNN
Cyprinidae	Barbinae	*Gymnodiptychus*	*pachycheilus*	AY463511	GCACTGGAATTAACC---N------------NNNNNNNNNN
Cyprinidae	Barbinae	*Lobocheilos*	sp	EF151091	GCATTGAAATGAGCC---T------------GCCCTAGTAG
Cyprinidae	Barbinae	*Paracrossochilus*	*vittatus*	EF151092	GCACTAGAATGAGCT---T------------GCCCTAGTAG
Cyprinidae	Barbinae	*Platypharodon*	*extremus*	AY463498	GCACTGGAATTAGCC---N------------NNNNNNNNNN
Cyprinidae	Barbinae	*Pseudobarbus*	*afer*	AF180851	GTGCTTAAACTAGCC---N------------NNNNNNNNNN
Cyprinidae	Barbinae	*Pseudobarbus*	*asper*	AF180850	GTGCTTAAACTAGCC---N------------NNNNNNNNNN
Cyprinidae	Barbinae	*Ptychobarbus*	*chungtienensis*	AY463508	GCACTACAATTAGCC---N------------NNNNNNNNNN
Cyprinidae	Barbinae	*Ptychobarbus*	*dipogon*	AY463510	GCACTACAATTAGCC---N------------NNNNNNNNNN
Cyprinidae	Barbinae	*Puntius*	*conchonius*	AY004751	ACACTAAAACTAGCT---T------------NNNNNNNNNN
Cyprinidae	Barbinae	*Puntius*	sp "Odessa"	EF151093	GCACTGAAACTAACT---T------------GCCCTAGTAG
Cyprinidae	Barbinae	*Puntius*	*titteya*	AF287455	ATTCTCCGACTCCCC---T------------NNNNNNNNNN
Cyprinidae	Barbinae	*Schizopygopsis*	*pylzovi*	AY463503	GCACTGGAATTAGCC---N------------NNNNNNNNNN
Cyprinidae	Barbinae	*Schizothorax*	*argentatus*	AF180861	GCACTAGAATGAGCT---N------------NNNNNNNNNN
Cyprinidae	Barbinae	*Schizothorax*	*waltoni*	AY463518	GCACTAGAATGAGCT---N------------NNNNNNNNNN
Cyprinidae	Barbinae	*Semilabeo*	*prochilus*	AF051881	GCACTAGAATGAGCC---N------------NNNNNNNNNN
Cyprinidae	Barbinae	*Sinocyclocheilus*	*oxycephalus*	AY854685	GCACTACAATGAGCT---N------------NNNNNNNNNN
Cyprinidae	Barbinae	*Sinocyclocheilus*	*rhinocerous*	AY854720	GCACTACAACTAGCT---N------------NNNNNNNNNN
Cyprinidae	Barbinae	*Sinocyclocheilus*	*yishanensis*	AB196444	GCACTACAATGAGCT---N------------NNNNNNNNNN
Cyprinidae	Barbinae	*Spinibarbichthys*	*sinensis*	AY195632	GCACTAGAATGAGCT---N------------NNNNNNNNNN
Cyprinidae	Barbinae	*Spinibarbus*	*caldwelli*	AY195627	ACACTAGAATGAGCT---N------------NNNNNNNNNN
Cyprinidae	Barbinae	*Spinibarbus*	*hollandi*	AY195629	ACGTTAGAGCAAACC---N------------NNNNNNNNNN
Cyprinidae	Barbinae	*Varicorhinus*	*mariae*	AF180863	GCACTGGAATTAACC---N------------NNNNNNNNNN
Cyprinidae	Barbinae	*Varicorhinus*	*steindachneri*	AF180865	GCACTGGAATTAACC---N------------NNNNNNNNNN
Cyprinidae	Cultrinae	*Chanodichthys*	*erythropterus*	AF051859	GCACTAAAATGAGCT---N------------NNNNNNNNNN
Cyprinidae	Cultrinae	*Hemiculter*	*leucisculus*	AF494362	GCACTAAAATGAGCT---T------------NNNNNNNNNN
Cyprinidae	Cultrinae	*Hemiculterella*	*macrolepis*	EF151094	GCACTGAAATGAGCT---T------------GCTCTAGTAG
Cyprinidae	Cultrinae	*Ischikauia*	*steenackeri*	AF375862	GCACTAAAATGAGCT---N------------NNNNNNNNNN
Cyprinidae	Cultrinae	*Megalobrama*	*pellegrini*	AF051869	GCACTAAAATGAGCT---N------------NNNNNNNNNN
Cyprinidae	Cultrinae	*Parabramis*	*pekinensis*	AF051874	GCACTAAAATGAGCT---N------------NNNNNNNNNN
Cyprinidae	Cyprininae	*Carassius*	*carassius*	NC006291	GCACTGAAATGAGCT---T------------NNNNNNNNNN
Cyprinidae	Cyprininae	*Cyprinus*	*carpio*	AB158807	GCACTAAAATGAGCT---T------------NNNNNNNNNN
Cyprinidae	Gobioninae	*Abbottina*	*rivularis*	AF051856	GCCATAAAATGAGCT---N------------NNNNNNNNNN
Cyprinidae	Gobioninae	*Biwia*	*zezera*	AF309507	GCACTAAAATGAGCT---N------------NNNNNNNNNN
Cyprinidae	Gobioninae	*Coreoleuciscus*	*splendidus*	DQ267433	GCATTAAAATGAGCT---T------------NNNNNNNNNN
Cyprinidae	Gobioninae	*Gobio*	*gobio*	AF045996	GCATTAAAATGAGCT---T------------NNNNNNNNNN

Cyprinidae	Gobioninae	*Gobiobotia*	*abbreviata*	AF051861	GCATTAAAATGAGCT---N------------NNNNNNNNNN
Cyprinidae	Gobioninae	*Gobiobotia*	*meridionalis*	AF375867	GCATTAAAATGAGCT---N------------NNNNNNNNNN
Cyprinidae	Gobioninae	*Gobiocypris*	*rarus*	AF309083	GCATTAAAATGAGCT---N------------NNNNNNNNNN
Cyprinidae	Gobioninae	*Hemibarbus*	*labeo*	DQ267432	GCACTAAAATGAGCT---T------------NNNNNNNNNN
Cyprinidae	Gobioninae	*Hemibarbus*	*longirostris*	DQ267422	GCACTAAAATGAGCT---T------------NNNNNNNNNN
Cyprinidae	Gobioninae	*Pseudogobio*	*vaillanti*	AY882923	GCACTAAAATGAGCT---N------------NNNNNNNNNN
Cyprinidae	Gobioninae	*Pseudorasbora*	*parva*	AF051873	GCATTAAAATGAGCT---N------------NNNNNNNNNN
Cyprinidae	Gobioninae	*Pungtungia*	*herzi*	AF375864	GCACTAAAATGAGCT---N------------NNNNNNNNNN
Cyprinidae	Gobioninae	*Romanogobio*	*banarescui*	AF090751	GCACTGAAATGAGCT---T------------NNNNNNNNNN
Cyprinidae	Gobioninae	*Sarcocheilichthys*	*variegatus*	AB054124	GCACTAAAATGAGCT---T------------NNNNNNNNNN
Cyprinidae	Gobioninae	*Saurogobio*	*dabryi*	AY245091	GCATTAAAATGAGCT---N------------NNNNNNNNNN
Cyprinidae	Gobioninae	*Squalidus*	*gracilis*	AF375866	GCACTAAAATGAGCT---N------------NNNNNNNNNN
Cyprinidae	Gobioninae	*Xenophysogobio*	*boulengeri*	AF375868	GCATTAAAATGAGCC---N------------NNNNNNNNNN
Cyprinidae	Leuciscinae	*Abramis*	*brama*	Y10441	GCATTGAAATGAGCC---N------------NNNNNNNNNN
Cyprinidae	Leuciscinae	*Acanthalburnus*	*microlepis*	AY026407	GCACTGAAATGAGCC---N------------NNNNNNNNNN
Cyprinidae	Leuciscinae	*Acanthobrama*	*terraesanctae*	AY026406	GCCCTGAAATGAGCC---N------------NNNNNNNNNN
Cyprinidae	Leuciscinae	*Acrossocheilus*	*yunnanensis*	AF051857	GCACTAGAATGAGCC---N------------NNNNNNNNNN
Cyprinidae	Leuciscinae	*Agosia*	*chrysogaster*	AF452081	ACATTGAAATGAGCT---N------------NNNNNNNNNN
Cyprinidae	Leuciscinae	*Alburnoides*	*bipunctatus*	Y10445	GCACTGAAATGAGCC---N------------NNNNNNNNNN
Cyprinidae	Leuciscinae	*Alburnus*	*alburnus*	Y10443	GCATTGAAATGAGCT---N------------NNNNNNNNNN
Cyprinidae	Leuciscinae	*Alburnus*	*chalcoides*	AY026394	GCATTGAAATGAGCT---N------------NNNNNNNNNN
Cyprinidae	Leuciscinae	*Alburnus*	*filippi*	AF095602	GCATTGAAATGAGCC---T------------NNNNNNNNNN
Cyprinidae	Leuciscinae	*Anaecypris*	*hispanica*	AF045978	GCATTGAAATGAGCC---T------------NNNNNNNNNN
Cyprinidae	Leuciscinae	*Aspius*	*aspius*	AY026398	GCATTAAAATGAGCC---N------------NNNNNNNNNN
Cyprinidae	Leuciscinae	*Aspius*	*vorax*	AY026399	GCATTGAAATGAGCC---N------------NNNNNNNNNN
Cyprinidae	Leuciscinae	*Blicca*	*bjoerkna*	Y10442	GCACTGAAATGAGCC---N------------NNNNNNNNNN
Cyprinidae	Leuciscinae	*Campostoma*	*anomalum*	DQ536421	GCACTGAAATGAGCT---T------------NNNNNNNNNN
Cyprinidae	Leuciscinae	*Chondrostoma*	*lemmingii*	DQ536427	GCGTTGAAATGAGCT---T------------NNNNNNNNNN
Cyprinidae	Leuciscinae	*Chondrostoma*	*nasus*	AF533760	GCATTGAAATGAGCC---T------------NNNNNNNNNN
Cyprinidae	Leuciscinae	*Chondrostoma*	*polylepis*	AF045982	GCATTGAAATGAGCC---T------------NNNNNNNNNN
Cyprinidae	Leuciscinae	*Codoma*	*ornata*	AY281060	GCGCTCAAA---------TAA----------GCCNNNNNNN
Cyprinidae	Leuciscinae	*Couesius*	*plumbeus*	AF452083	GCACTGAAATGAGCC---N------------NNNNNNNNNN
Cyprinidae	Leuciscinae	*Ctenopharyngodon*	*idella*	AF051860	GCACTAAAATGAGCT---N------------NNNNNNNNNN
Cyprinidae	Leuciscinae	*Cyprinella*	*galactura*	AY249538	GCCCTGAAANNNNNN---N------------NNNNNNNNNN
Cyprinidae	Leuciscinae	*Cyprinella*	*spiloptera*	U66605	GCCCTGAAATGAGCC---N------------NNNNNNNNNN
Cyprinidae	Leuciscinae	*Elopichthys*	*bambusa*	AY744501	ATACTAGAAAAACAT---N------------NNNNNNNNNN
Cyprinidae	Leuciscinae	*Eremichthys*	*acros*	AF370117	GCATTAAAATGAGCT---N------------NNNNNNNNNN
Cyprinidae	Leuciscinae	*Ericymba*	*buccata*	AF117154	GCGCTGAAATGAGCT---N------------NNNNNNNNNN
Cyprinidae	Leuciscinae	*Erimystax*	*cahni*	AY486010	GNNNNNNNNNNNNNN---N------------NNNNNNNNNN
Cyprinidae	Leuciscinae	*Erimystax*	*dissimilis*	AY486027	GNNNNNNNNNNNNNN---N------------NNNNNNNNNN
Cyprinidae	Leuciscinae	*Gila*	*cypha*	AF452074	GCACTGAAATGAGCT---N------------NNNNNNNNNN
Cyprinidae	Leuciscinae	*Gila*	*orcuttii*	AF370118	GCACTGAAATGAGCT---N------------NNNNNNNNNN
Cyprinidae	Leuciscinae	*Gila*	*robusta*	DQ536424	GCACTGAAATGAGCT---T------------NNNNNNNNNN
Cyprinidae	Leuciscinae	*Hemitremia*	*flammea*	AY281054	GCATTGGGATTTACC---N------------NNNNNNNNNN
Cyprinidae	Leuciscinae	*Hesperoleucus*	*symmetricus*	AF370116	GCACTAAAATGAGCT---N------------NNNNNNNNNN
Cyprinidae	Leuciscinae	*Hybognathus*	*hankinsoni*	AF452080	GCACTGAAATGAGCC---N------------NNNNNNNNNN
Cyprinidae	Leuciscinae	*Hybopsis*	*winchelli*	AF117164	GCGTTGAAATGAGCT---N------------NNNNNNNNNN
Cyprinidae	Leuciscinae	*Iotichthys*	*phlegethontis*	AY641427	NNNNNNNNNNNNNNN---N------------NNNNNNNNNN
Cyprinidae	Leuciscinae	*Ladigesocypris*	*ghigii*	AJ251091	GCATTAAAATGAGCC---N------------NNNNNNNNNN
Cyprinidae	Leuciscinae	*Lepidomeda*	*albivallis*	AF452089	GCACTGAAATGAGCC---N------------NNNNNNNNNN
Cyprinidae	Leuciscinae	*Squalidus*	*cephalus*	AF045995	GCATTGAAATGAACC---T------------NNNNNNNNNN
Cyprinidae	Leuciscinae	*Leuciscus*	*leuciscus*	AY509823	GCATTGGAATGAGCC---N------------NNNNNNNNNN
Cyprinidae	Leuciscinae	*Squalidus*	*pyrenaicus*	AF045991	GCATTGAAATGAGCT---T------------NNNNNNNNNN
Cyprinidae	Leuciscinae	*Luxilus*	*albeolus*	U66598	GCACTGAAATGAGCT---N------------NNNNNNNNNN
Cyprinidae	Leuciscinae	*Lythrurus*	*fumeus*	U17269	GCATTGAAATGAGCT---T------------NNNNNNNNNN
Cyprinidae	Leuciscinae	*Lythrurus*	*lirus*	U17273	GCGTTGAAATGAGCT---T------------NNNNNNNNNN
Cyprinidae	Leuciscinae	*Margariscus*	*margarita*	AF452072	GCACTTAAATGAGCT---N------------NNNNNNNNNN
Cyprinidae	Leuciscinae	*Meda*	*fulgida*	AF452094	GCATTGAAATGAGCC---N------------NNNNNNNNNN
Cyprinidae	Leuciscinae	*Moapa*	*coriacea*	AF452075	ACACTGGAATGAGCT---N------------NNNNNNNNNN

Cyprinidae	Leuciscinae	*Mylocheilus*	*caurinus*	AF117168	GCACTGAAATGAGCT---N------------NNNNNNNNNN
Cyprinidae	Leuciscinae	*Nocomis*	*biguttatus*	AY486057	GNNNNNNNNNNNNNN---N------------NNNNNNNNNN
Cyprinidae	Leuciscinae	*Nocomis*	*micropogon*	AF452077	GCACTGAAGTGAGCT---N------------NNNNNNNNNN
Cyprinidae	Leuciscinae	*Notemigonus*	*crysoleucas*	U01318	GCATTGAAATGAGCT---T------------NNNNNNNNNN
Cyprinidae	Leuciscinae	*Notropis*	*stramineus*	DQ536429	GCCCTGAAATGAGCT---T------------NNNNNNNNNN
Cyprinidae	Leuciscinae	*Notropis*	*telescopus*	AF352289	GCCCTGAAATGAGCC---N------------NNNNNNNNNN
Cyprinidae	Leuciscinae	*Notropis*	*texanus*	AF352267	GCATTAAAATGAGCT---N------------NNNNNNNNNN
Cyprinidae	Leuciscinae	*Ochetobius*	*elongatus*	AF309506	GCACTAAAATGAGCT---N------------NNNNNNNNNN
Cyprinidae	Leuciscinae	*Opsopoeodus*	*emilae*	U17270	GCGTTAGAA---------TAA----------GCTTNNNNNN
Cyprinidae	Leuciscinae	*Orthodon*	*microlepidotus*	AF452073	GCGTTGAAATGAGCT---N------------NNNNNNNNNN
Cyprinidae	Leuciscinae	*Pachychilon*	*pictum*	AF090762	GCATTGAAATGAGCT---T------------NNNNNNNNNN
Cyprinidae	Leuciscinae	*Pelecus*	*cultratus*	AY838938	GCATTAAAATGAGCT---T------------NNNNNNNNNN
Cyprinidae	Leuciscinae	*Phenacobius*	*catostomus*	AY486055	GNNNNNNNNNNNNNN---N------------NNNNNNNNNN
Cyprinidae	Leuciscinae	*Phenacobius*	*mirabilis*	DQ536431	GCACTAAAATGAGCT---T------------NNNNNNNNNN
Cyprinidae	Leuciscinae	*Phenacobius*	*uranops*	AY486056	GNNNNNNNNNNNNNN---N------------NNNNNNNNNN
Cyprinidae	Leuciscinae	*Phoxinellus*	*alepidotus*	AY494746	GCATTGAAATGAGCC---T------------NNNNNNNNNN
Cyprinidae	Leuciscinae	*Phoxinellus*	*dalmaticus*	AY494742	GCATTGAAATGAGCC---T------------NNNNNNNNNN
Cyprinidae	Leuciscinae	*Phoxinus*	*phoxinus*	Y1044	ACATTAAAATGAGCC---N------------NNNNNNNNNN
Cyprinidae	Leuciscinae	*Pimephales*	*notatus*	U66606	GCATTGAAATGAGCT---N------------NNNNNNNNNN
Cyprinidae	Leuciscinae	*Pimephales*	*vigilax*	AF117202	GCATTGAAATGAGCT---N------------NNNNNNNNNN
Cyprinidae	Leuciscinae	*Plagopterus*	*argentissimus*	AF452090	GCATTGAAATGAGCC---N------------NNNNNNNNNN
Cyprinidae	Leuciscinae	*Pogonichthys*	*macrolepidotus*	AY096009	GCACTGAAATGAGCT---N------------NNNNNNNNNN
Cyprinidae	Leuciscinae	*Pseudaspius*	*leptocephalus*	AB162649	GCATTGAAATGAGCT---N------------NNNNNNNNNN
Cyprinidae	Leuciscinae	*Pseudophoxinus*	*anatolicus*	AY494754	GCATTGAAATGAGCC---T------------NNNNNNNNNN
Cyprinidae	Leuciscinae	*Pseudophoxinus*	*crassus*	AY494763	GCATTGAAATGAGCC---T------------NNNNNNNNNN
Cyprinidae	Leuciscinae	*Pteronotropis*	*hubbsi*	AF261224	GCCCTAAAATGAGCT---T------------NNNNNNNNNN
Cyprinidae	Leuciscinae	*Relictus*	*solitarius*	AF370115	GCACTAAAATGAGCT---N------------NNNNNNNNNN
Cyprinidae	Leuciscinae	*Rhinichthys*	*atratulus*	AF452078	GCATTGAAATGAGCT---N------------NNNNNNNNNN
Cyprinidae	Leuciscinae	*Richardsonius*	*balteatus*	AY096011	GCATTGAAATGAGCT---N------------NNNNNNNNNN
Cyprinidae	Leuciscinae	*Rutilus*	*rutilus*	AF090772	GCATTGAAATGAGCC---T------------NNNNNNNNNN
Cyprinidae	Leuciscinae	*Scardinius*	*acarnanicus*	AF090775	GCATTGAAATGAGCC---T------------NNNNNNNNNN
Cyprinidae	Leuciscinae	*Scardinius*	*erythrophthalmus*	AY509848	GCATTGGAATGGCCC---N------------NNNNNNNNNN
Cyprinidae	Leuciscinae	*Semotilus*	*atromaculatus*	AF452082	GCACTGAAATGAGCT---N------------NNNNNNNNNN
Cyprinidae	Leuciscinae	*Siphateles*	*alvordensis*	AF370041	GCACTGAAATGAGCT---N------------NNNNNNNNNN
Cyprinidae	Leuciscinae	*Siphateles*	*bicolor*	AF237751	GCACTGAAATGAGCT---N------------NNNNNNNNNN
Cyprinidae	Leuciscinae	*Snyderichthys*	*copei*	AF270914	GCACTGAAATGAGCC---N------------NNNNNNNNNN
Cyprinidae	Leuciscinae	*Squalius*	*albus*	AY549460	GCACTGAAATGAGCC---T------------NNNNNNNNNN
Cyprinidae	Leuciscinae	*Squalius*	*palaciosi*	AF045990	GCATTGAAATGAGCT---T------------NNNNNNNNNN
Cyprinidae	Leuciscinae	*Telestes*	*beoticus*	AF090770	GCATTGAAATGAGCC---T------------NNNNNNNNNN
Cyprinidae	Leuciscinae	*Telestes*	*souffia*	AY509862	GCATTGAAATGAGCC---N------------NNNNNNNNNN
Cyprinidae	Leuciscinae	*Tribolodon*	*hakonensis*	AB162647	GCACTGAAATGAGCT---N------------NNNNNNNNNN
Cyprinidae	Leuciscinae	*Tribolodon*	*nakamurai*	AB162648	GCACTGAAATGAGCT---N------------NNNNNNNNNN
Cyprinidae	Leuciscinae	*Tropidophoxinellus*	*hellenicus*	AF090776	GCATTGAAATGAGCC---T------------NNNNNNNNNN
Cyprinidae	Leuciscinae	*Tropidophoxinellus*	*spartiaticus*	AF090777	GCATTGAAATGAGCC---T------------NNNNNNNNNN
Cyprinidae	Leuciscinae	*Vimba*	*vimba*	AY026404	GCATTGAAATGAGCC---N------------NNNNNNNNNN
Cyprinidae	Leuciscinae	*Yuriria*	*alta*	AF469163	GCATTGAAATGAGCT---T------------NNNNNNNNNN
Cyprinidae	Rasborinae	*'Danio'*	*erythromicron*	EF151095	GCACTAAAATGGGCC---T------------GCCCTAGTAG
Cyprinidae	Rasborinae	*Aphyocypris*	*chinensis*	AF307452	GCACTAAAATGAGCT---N------------NNNNNNNNNN
Cyprinidae	Rasborinae	*Boraras*	*maculatus*	EF151096	CTTCTAGGT---------TAGACCT------GCCTTAATAG
Cyprinidae	Rasborinae	*Candidia*	*barbata*	AY958200	GCACTAAAATGAGCT---N------------NNNNNNNNNN
Cyprinidae	Rasborinae	*Chela*	*dadiburjori*	EF151097	ACTCTAAAACAAGCC---T------------GCGCTAGTAG
Cyprinidae	Rasborinae	*Chela*	*maasii*	EF151098	GCACTTGAATGAGAA---T------------GCCCTAGTAG
Cyprinidae	Rasborinae	*Danio*	*rerio*	NC002333	GCACTCCAATGAAGC---T------------NNNNNNNNNN
Cyprinidae	Rasborinae	*Danionella*	sp "Myanmar"	EF151099	ACACTCAAATGAGAAAACT------------GCCCTAGTAG
Cyprinidae	Rasborinae	*Devario*	*regina*	EF151100	AACCTAAAACTAACC---T------------GCCCTAGTAG
Cyprinidae	Rasborinae	*Esomus*	*metallicus*	EF151101	ACAGCCCACCAAACC---T------------GCTTCGGTAG
Cyprinidae	Rasborinae	*Hemigrammocypris*	*rasborella*	AF375863	GCACTAAAATGAGCT---N------------NNNNNNNNNN
Cyprinidae	Rasborinae	*Horadandia*	*atukorali*	EF151102	ACACTGTAT---------TAAGCCT------GCCTTAGTAG
Cyprinidae	Rasborinae	*Inlecypris*	*auropurpureus*	EF151103	AACCTGAAACTAGCT---T------------GCCCTAGTAG

Cyprinidae	Rasborinae	*Kosswigobarbus*	*kosswigi*	AF180853	ACACTAGAATTAGCT---N------------NNNNNNNNNN
Cyprinidae	Rasborinae	*Luciosoma*	sp	EF151104	GCACTTGAATGAGAC---T------------GCCCTAGTAG
Cyprinidae	Rasborinae	*Microrasbora*	*kubotai*	EF151105	ACATTAAGCCGCACA---T------------GCCCTAGTAG
Cyprinidae	Rasborinae	*Nematabramis*	*steindachneri*	EF151106	GCACTTGAATGAGAC---T------------GCCCTAGTAG
Cyprinidae	Rasborinae	*Opsariichthys*	*bidens*	AY245090	GCACTGAAATGAGCT---N------------NNNNNNNNNN
Cyprinidae	Rasborinae	*Opsariichthys*	sp	EF151107	GCATTAAAATGAGCT---T------------GCCCTAGTAG
Cyprinidae	Rasborinae	*Opsariichthys*	*uncirostris*	AF308437	GCACTGAAATGAGCT---N------------NNNNNNNNNN
Cyprinidae	Rasborinae	*Opsariichthys*	*pachycephalus*	AY958189	GCATTGAAATGAGCT---N------------NNNNNNNNNN
Cyprinidae	Rasborinae	*Paedocypris*	sp "Banka"	EF151108	ATACTTAAA---------TAATA--------GCATTAGTAG
Cyprinidae	Rasborinae	*Paedocypris*	sp "Kalimantan Tengah"	EF151109	ATACTTAAC---------T------------GCATTAGTAG
Cyprinidae	Rasborinae	*Paedocypris*	sp "Pontianak"	EF151110	ATACTTAAC---------T------------GCATTAGTAG
Cyprinidae	Rasborinae	*Paedocypris*	sp "Pulau Singkep"	EF151111	ATACTTAAA---------TAATA--------GCATTAGTAG
Cyprinidae	Rasborinae	*Parachela*	sp	EF151112	GCACTAAAATGAGCT---T------------GCCCTAGTAG
Cyprinidae	Rasborinae	*Parazacco*	*fasciatus*	AY958195	GCATTAAAATGAGCT---N------------NNNNNNNNNN
Cyprinidae	Rasborinae	*Pectenocypris*	*korthausae*	EF151113	CACTGGATA---------TAAGCC-------GCTTTAGTAG
Cyprinidae	Rasborinae	*Rasbora*	*cephalotaenia*	EF151114	GCATTAGACCGGGCC---T------------GCCTTAGTAG
Cyprinidae	Rasborinae	*Rasbora*	*daniconius*	EF151115	GCACTAGATTGAGCC---T------------GCCTTAGTAG
Cyprinidae	Rasborinae	*Rasbora*	*kalbarensis*	EF151116	GCACTAGCT---------TAGATC-------GCCTTAGTAG
Cyprinidae	Rasborinae	*Rasbora*	*kalochroma*	EF153103	ACA---------------TAAAATTTAGCCCGCCCTAGTAG
Cyprinidae	Rasborinae	*Rasbora*	*pauciperforata*	EF151117	GTAACATAT---------TAAGCCT------GCCTCAGTAG
Cyprinidae	Rasborinae	*Rasbora*	*vulcanus*	EF151118	AAACTAGACCAGGGC---T------------GCCTTAGTAG
Cyprinidae	Rasborinae	*Sundadanio*	*axelrodi*	EF151119	GCACTTAACTGAAAC---T------------GCTCTAGTAG
Cyprinidae	Rasborinae	*Sundadanio*	*axelrodi*	EF151120	GCACTTAACTGAAAC---T------------GCCCTAGTAG
Cyprinidae	Rasborinae	*Tanichthys*	*albonubes*	EF151121	GCATTAAAATGAGCC---T------------GCCCTAGTAG
Cyprinidae	Rasborinae	*Tanichthys*	*albonubes*	AF375869	AACCTAAAATGAGCT---N------------NNNNNNNNNN
Cyprinidae	Rasborinae	*Trigonostigma*	*heteromorpha*	EF151122	GCACTAAAC---------TAGAACT------GTCCTAGTAG
Cyprinidae	Rasborinae	*Yaoshanicus*	*arcus*	AF309086	GCACTAAAATGAGCT---N------------NNNNNNNNNN
Cyprinidae	Rasborinae	*Zacco*	*platypus*	AF309085	GCATTGAAATGAGCT---N------------NNNNNNNNNN
Cyprinidae	Squaliobarbinae	*Mylopharyngodon*	*piceus*	AF051870	GCACTAAAATGAGCT---N------------NNNNNNNNNN
Cyprinidae	Squaliobarbinae	*Squaliobarbus*	*curriculus*	AF051877	GCACTAAAATGAGCT---N------------NNNNNNNNNN
Cyprinidae	Tincinae	*Tinca*	*tinca*	Y10451	GCATTGAAATGAACT---N------------NNNNNNNNNN
Cyprinidae	Xenocyprinae	*Distoechodon*	*compressus*	AF374407	GCATTAAAATGAGCT---N------------NNNNNNNNNN
Cyprinidae	Xenocyprinae	*Hypophthalmichthys*	*molitrix*	AF051866	GCACTAAAATGAGCT---N------------NNNNNNNNNN
Cyprinidae	Xenocyprinae	*Hypophthalmichthys*	*nobilis*	AF051855	GCACTAAAATGAGCT---N------------NNNNNNNNNN
Cyprinidae	Xenocyprinae	*Pseudobrama*	*simoni*	AF036194	GCATTAAAATGAGCT---N------------NNNNNNNNNN
Cyprinidae	Xenocyprinae	*Xenocyprioides*	*carinatus*	AF036201	GCACTAAAATGAGCT---N------------NNNNNNNNNN
Cyprinidae	Xenocyprinae	*Xenocyprioides*	*parvulus*	AF036207	GCACTACAATGAGCT---N------------NNNNNNNNNN
Cyprinidae	Xenocyprinae	*Xenocypris*	*fangi*	AF036205	GCATTAAAATGAGCT---N------------NNNNNNNNNN
Cyprinidae	unassigned	*Acrocheilus*	*alutaceus*	AF452076	GCACTGAAATGAGCT---N------------NNNNNNNNNN
other					
Balitoridae		*Barbatula*	*barbatula*	AY281267	GCCCTAGAATGAGCT---T------------NNNNNNNNNN
Balitoridae		*Gastromyzon*	*ctenocephalus*	AY281272	GCACTAGAATGAGCT---T------------NNNNNNNNNN
Balitoridae		*Hemimyzon*	*formosanum*	AY281275	GCACTTGAATGAGCC---T------------NNNNNNNNNN
Balitoridae		*Schistura*	*longa*	AY625698	GCCCTAGAATGAGCT---N------------NNNNNNNNNN
Catostomidae		*Carpiodes*	*carpio*	AB126083	GCACTAGAATGAGCC---T------------NNNNNNNNNN
Catostomidae		*Catostomus*	*catostomus*	AF454871	GCACTGGAATGAGCT---N------------NNNNNNNNNN
Catostomidae		*Moxostoma*	*anisurum*	AF454880	GCACTTGAATGAGCT---N------------NNNNNNNNNN
Catostomidae		*Myxocyprinus*	*asiaticus*	AB223007	GCACTAGAATGAGCT---T------------NNNNNNNNNN
Catostomidae		*Scartomyzon*	*congestus*	AF180820	GCACTTGAATGAGCT---N------------NNNNNNNNNN
Cobitidae		*Leptobotia*	*elongata*	AY281264	GCACTAGAATGAGCC---T------------NNNNNNNNNN
Cobitidae		*Misgurnus*	*anguillicaudatus*	AF051868	GCATTAGAGTGAGCT---N------------NNNNNNNNNN
Cobitidae		*Sabanejewia*	*aurata*	AF499190	GCATTAGAATGAGCT---N------------NNNNNNNNNN
Gyrinocheilidae		*Gyrinocheilus*	*aymonieri*	DQ105256	GCACTAGAATGAGCT---N------------NNNNNNNNNN
Gyrinocheilidae		*Gyrinocheilus*	*pustulosus*	EF151123	GCACTAGAATGAGCT---T------------GCCCTAGTAG
Outgroup					
Chanidae (Gonorynchiformes)		*Chanos*	*chanos*	NC4693	GCCCTTGAATGAGCC---T------------NNNNNNNNNN
