# Supplementary material for: Evolution of miniaturization and the phylogenetic position of Paedocypris, comprising the world's smallest vertebrate
Source: BMC Evol Biol. 2007 Mar 13;7:38. doi: 10.1186/1471-2148-7-38 (PMC1838906; doi:10.1186/1471-2148-7-38)
Supplement: Additional File 2 — Table 2 – Additional primers used to amplify the complete cytb of the taxa sequenced specifically for this study. This table lists the additional primers used for this study. For previously published primers the references are given, primers designed for this study are given 5' to 3'. [file 1471-2148-7-38-S2.PDF]

Additional file 2.

In cases where it was not possible to amplify the complete cytb with the primers DonGlu F and DonThr R [1] the cyt*b* was amplified in two overlapping fragments (all primers are given 3' – 5'):

3' half of cyt*b*
combinations of these forward and reverse primers

DonGlu F	[1]
MNCN-Glu F	[2]
LR_GluF1	GTTGTAGTTCAACTACAAGAAC
LR_GluF3	TGACTCGAAAAACCACTGTTGT
LR_cytb605R	GGRTTRTTWGAYCCNGTTT
LR_cytb679R	GAANCCWAGNAGRTCYTT
LR_cytb739b	TCYGGRTCNCCTARNARRTTNGG

5' half of the cyt*b*
combinations of these forward and reverse primers

CypInt529F	CGRTTYTTCGCMTTYCAYTT
DonThr R	[1]
FishProR	AGTTTAATTTAGAATYTTRGCTTTGG

1. Rüber L, Britz R, Tan HH, Ng PKL, Zardoya R: **Evolution of mouthbrooding and life-history correlates in the fighting fish genus Betta**. *Evolution* 2004, **58**(4):799-813.
2. San Mauro D, Gower DJ, Oommen OV, Wilkinson M, Zardoya R: **Phylogeny of caecilian amphibians (Gymnophiona) based on complete mitochondrial genomes and nuclear RAG1**. *Molecular Phylogenetics and Evolution* 2004, **33**(2):413-427.
